# Reporter cell lines to evaluate the selectivity of chemicals for human and zebrafish estrogen and peroxysome proliferator activated γ receptors

**DOI:** 10.3389/fnins.2015.00212

**Published:** 2015-06-09

**Authors:** Marina Grimaldi, Abdelhay Boulahtouf, Vanessa Delfosse, Erwan Thouennon, William Bourguet, Patrick Balaguer

**Affiliations:** ^1^Institut de Recherche en Cancérologie de MontpellierMontpellier, France; ^2^Institut National de la Santé et de la Recherche Médicale U1194Montpellier, France; ^3^Université MontpellierMontpellier, France; ^4^Institut Reìgional du Cancer de MontpellierMontpellier, France; ^5^Institut National de la Santé et de la Recherche Médicale U1054Montpellier, France; ^6^Centre National de la Recherche Scientifique UMR5048, Centre de Biochimie Structurale, Université MontpellierMontpellier, France

**Keywords:** estrogen receptor, peroxysome proliferator activated receptor γ, environmental disrupting compounds, reporter cell lines, human, zebrafish

## Abstract

Zebrafish is increasingly used as an animal model to study the effects of environmental nuclear receptors (NRs) ligands. As most of these compounds have only been tested on human NRs, it is necessary to measure their effects on zebrafish NRs. Estrogen receptors (ER) α and β and peroxysome proliferator activated receptor (PPAR) γ are main targets of environmental disrupting compounds (EDCs). In humans there are two distinct nuclear ERs (hERα and hERβ), whereas the zebrafish genome encodes three ERs, zfERα, zfERβ1, and zfERβ2. Only one isoform of PPARγ is expressed in both humans and zebrafish. In this review, we described reporter cell lines that we established to study the interaction of EDCs with human and zebrafish ERs and PPARγ. Using these cell lines, we observed that zfERs are thermo-sensitive while zfPPARγ is not. We also showed significant differences in the ability of environmental and synthetic ligands to modulate activation of zfERs and zfPPARγ in comparison to hERs and hPPARγ. Some environmental estrogens (bisphenol A, mycoestrogens) which are hER panagonists displayed greater potency for zfERα as compared to zfERβs. hERβ selective agonists (8βVE2, DPN, phytoestrogens) also displayed zfERα selectivity. Among hERα selective synthetic agonists, 16α-LE2 was the most zfERα selective compound. Almost all zfPPARγ environmental ligands (halogenated bisphenol A derivatives, phthalates, perfluorinated compounds) displayed similar affinity for human and zebrafish PPARγ while pharmaceutical hPPARγ agonists like thiazolidones are not recognized by zfPPARγ. Altogether, our studies show that all hERs and hPPARγ ligands do not control in a similar manner the transcriptional activity of zfERs and zfPPARγ and point out that care has to be taken in transposing the results obtained using the zebrafish as a model for human physiopathology.

## Introduction

Human nuclear hormone receptors (NHRs) are a family of 48 transcription factors, many of which have been shown to be activated by ligands. NHRs regulate cognate gene networks involved in key physiological functions such as cell growth and differentiation, development, homeostasis, or metabolism (Gronemeyer et al., 2004; Germain et al., 2006). Consequently, inappropriate exposure to environmental pollutants often leads to proliferative, reproductive, and metabolic diseases, including hormonal cancers, infertility, obesity or diabetes. NHRs are modular proteins composed of several domains, most notably an N-terminal domain, which harbors a ligand-independent activation function (AF-1), a central DNA-binding domain (DBD), and a C-terminal ligand-binding domain (LBD) hosting a ligand-dependent transcriptional activation function (AF-2) (Gronemeyer et al., 2004). In the absence of the cognate ligand, some NHRs are located in the nucleus, bind to the DNA response elements of their target genes, and recruit corepressors, while others are located in the cytoplasm in an inactive complex with chaperones.

Ligand binding induces major structural alterations of the receptor LBDs, leading to (1) destabilization of corepressor or chaperone interfaces, (2) exposure of nuclear localization signals to allow nuclear translocation and DNA binding of cytoplasmic receptors, and (3) recruitment of coactivators triggering gene transcription through chromatin remodeling and activation of the general transcription machinery. The crystal structures of many NHR LBDs have been determined, revealing a conserved core of 12 α-helices (H1–H12) and a short two-stranded antiparallel β-sheet (S1 and S2) arranged into a three-layered sandwich fold. This arrangement generates a mostly hydrophobic cavity in the lower half of the domain, which can accommodate the cognate ligand. In all hormone-bound LBD structures, the ligand-binding pocket (LBP) is sealed by helix H12. This conformation is specifically induced by the binding of hormones or synthetic agonists and is referred to as the “active conformation” because it allows the dissociation of corepressors and favors the recruitment of transcriptional coactivators (Bourguet et al., 2000; Renaud and Moras, 2000; Pike, 2006).

In contrast to agonist binding, interaction with antagonists prevents the correct positioning of helix H12, thus avoiding association with the LxxLL motifs of coactivators. The LBD also contributes to the modulation of the N-terminal AF-1 through interdomain crosstalk so that both AF-1 and AF-2 domains can recruit a range of coregulatory proteins and act individually or in a synergistic manner (Benecke et al., 2000; Bommer et al., 2002; Wilson, 2011).

Among nuclear receptors, ERs and PPARγ are main targets of numerous synthetic substances released into the environment by human activities. These substances can act as endocrine-disrupting chemicals (EDCs) causing reproductive, developmental, metabolic, or neurological diseases as well as hormone-related cancers (Diamanti-Kandarakis et al., 2009). Many EDCs are man-made compounds, for example bisphenols, phthalates, parabens, dioxins, pesticides, alkylphenols, organotins, polychlorinated biphenyls, or perfluoroalkyl compounds. Some natural EDCs can also be found in plants and fungi. Standard methods to study interaction of EDCs with these nuclear receptors use stable cell reporter gene assays based on human ERs and PPARγ activation (Balaguer et al., 1999; Legler et al., 1999; Seimandi et al., 2005; Riu et al., 2011a). To address whether chemicals exert an effect at the organismal level, ER activity assays have been developed for zebrafish. In these animals, GFP reporter constructs are designed to act in certain tissues exclusively (such as liver or brain) (Kurauchi et al., 2005; Brion et al., 2012) or in all tissues of embryos and larvae (Gorelick and Halpern, 2011; Lee et al., 2012). Zebrafish has also been used as an *in vivo* model to study the effect of environmental compounds on PPARγ (Riu et al., 2014). Zebrafish stores neutral lipid triglycerides in visceral, intramuscular, and subcutaneous adipocyte depots (Tingaud-Sequeira et al., 2012). Studies of the zebrafish embryo, which is optically transparent thus facilitating the labeling and detection of lipid depots using lipid staining (Minchin and Rawls, 2011), have shown that white adipose tissue appearance is correlated with size rather than the age of the fish. By using zebrafish as a PPARγ ligand screening model, we have showed that halogenated-BPA analogs are potent inducers of lipid accumulation *in vivo* through PPARγ signaling (Riu et al., 2014).

In order to evaluate the effects of environmental and pharmaceutical compounds on the transcriptional activity of zfERs and zfPPARγ and to compare the data with their activity on hERs and hPPARγ, we established human and zebrafish ERs and PPARγ reporter cell lines in the same cellular context (Balaguer et al., 1999; Seimandi et al., 2005; Pinto et al., 2014; Riu et al., 2014). In HeLa cells stably expressing an ERE-driven luciferase reporter (HELN cells), we expressed the full-length hERα, hERβ, zfERα, zfERβ1, and zfERβ2, respectively. Similarly, in HeLa cells stably expressing a GAL4RE-driven luciferase reporter (HG5LN cells), we expressed a fusion protein consisting of the hPPARγ or zfPPARγ ligand binding domain (LBD) and the DNA binding domain (DBD) of the yeast transcription factor GAL4 (GAL4-PPARγ).

The resulting HELN-ERs and HG5LN PPARγ cell lines were used to evaluate the effects of environmental compounds on gene transactivation by the five ERs and the two PPARγ, and to compare these effects with results obtained on hER and PPARγ orthologs. Since zebrafish is used as a model for studying the effects of environmental compounds *in vivo*, determining the transcriptional profiles of these compounds on the zfERs and zfPPARγ is crucial to support the zebrafish model for ER- and PPARγ-related studies and their extrapolation to the mammalian system.

## Estrogen receptors

Estrogen signaling is mainly mediated by the two estrogen receptors ERα (also called NR3A1) and ERβ (also called NR3A2) (Jensen and Jordan, 2003; Dahlman-Wright et al., 2006) which play important roles in the growth and maintenance of various tissues such as the mammary gland, uterus, bones, or the cardiovascular system. Like most NRs, ERs bind as dimers to DNA response elements in the promoter region of target genes and respond to the naturally occurring sex hormone 17β-estradiol (E_2_). Both hERs are widely distributed throughout the body, displaying distinct but overlapping expression patterns in a variety of tissues (Couse and Korach, 1999). hERα is primarily expressed in the uterus, liver, kidney, and heart, whereas hERβ is preferentially expressed in the ovary, prostate, lung, gastrointestinal tract, bladder, and hematopoietic and central nervous systems (Kuiper et al., 1997). However, hERα and hERβ are coexpressed in a number of tissues including the mammary gland, thyroid, adrenal, bones, and some regions of the brain. Although hERα and hERβ share similar mechanisms of action, several differences in the transcriptional abilities of each receptor and distinct phenotypes between gene-null animals have been identified, suggesting that these receptors may regulate distinct cellular pathways (Curtis et al., 1996; Couse and Korach, 1999). Interestingly, when hERs are coexpressed, hERβ exhibits an inhibitory action on ERα-mediated gene expression (Pettersson et al., 2000; Liu et al., 2002), so that hERβ has been shown to antagonize several hERα-mediated effects including fat reduction and cell proliferation in breast, uterus, or prostate (Ogawa et al., 1998; Weihua et al., 2000; Lindberg et al., 2003). Furthermore, in addition to controlling the normal development and function of the reproductive system and other tissues, estrogens are key regulators of primary breast and prostatic cancer growth (Jensen and Jordan, 2003). Roughly 40% of human cancers require steroid hormones for their growth and the first-line therapy for treatment of hormone-dependent cancers is based on androgen and estrogen antagonists interacting with AR or ERs and shutting down the corresponding hormone-responsive pathway. Interestingly, ERβ has been shown to antagonize ERα-mediated effects on cell proliferation in the breast, uterus, ovary, and prostate (Weihua et al., 2000; Lindberg et al., 2003; Ellem and Risbridger, 2009). In this regard, estrogens with selectivity for either ER subtypes may produce different biological outcomes, particularly on cancer cell proliferation. Given the widespread role of ERs in human physiology, it is not surprising that environmental compounds which bind to ERs, thus substituting for the natural hormone and deregulating the fine-tuned action of E_2_, can lead to ER-related disorders including breast, endometrial, colorectal, or prostate cancers, as well as neurodegenerative, inflammatory, immune, cardiovascular, and metabolic diseases.

Small fish including zebrafish (*Danio rerio*) are increasingly being used as model species to study *in vivo* effects of EDCs (Segner, 2009; Vosges et al., 2010; Brion et al., 2012). In zebrafish, three zfER subtypes (zfERα, zfERβ1, and zfERβ2) are present (Menuet et al., 2002; Hawkins and Thomas, 2004). Zebrafish ERα (esr1) is orthologous to the human ERα, while ERβ1 (esr2b) and ERβ2 (esr2a) are orthologs of the human ERβ (Bardet et al., 2002). The overall amino-acid sequence identity between the zfER subtypes and their corresponding human ER orthologs is approximately 50% (Menuet et al., 2002). ZfERs are differently expressed and regulated in reproductive tissue like gonads, liver, as well as in brain. In adult liver, E2 induces zfERα expression while it has no effect on zfERβ2 and represses zfERβ1 expression (Menuet et al., 2002). Moreover, both zfERα and zfERβ2 upregulate zfERα expression after E2 exposure, whereas zfERβ1 has no effect on this expression (Menuet et al., 2004). These studies suggest that the different forms of zfERs have partially distinct and nonredundant functions. Hence, in the perspective of developing fish *in vitro* assays, it is essential to take into account all zfER subtypes in the assessment of chemical estrogenicity in zebrafish. Since these three zfERs are thought to mediate different biological effects, there is an increased interest in finding subtype-selective zfER ligands.

## Estrogen receptors reporter cell lines

To understand and to evaluate impact of xenoestrogens on ER-signaling pathway, it is necessary to develop cell-based transcription assay systems that could reflect different cellular contexts and/or different model species. *In vitro* assays based on reporter gene driven by ERE have been proven to be useful and relevant screening tools to address the large number of chemicals yet needed to be tested for their estrogenic potential. We and other groups have developed stable reporter gene assays based on human ERα and ERβ activation in different cell contexts and successfully used them to characterize estrogenic potency of chemicals (Balaguer et al., 1999; Legler et al., 1999; Wilson et al., 2004; Sotoca et al., 2008; Docquier et al., 2013). In order to take into account the species of origin of studied receptor in hazard assessment of estrogenic chemicals in fish, we have developed *in vitro* stable reporter gene assays derived from fish species (Molina-Molina et al., 2008; Cosnefroy et al., 2012; Pinto et al., 2014). Among them, HELN-zfERα, -zfERβ1, and -zfERβ2 (Pinto et al., 2014) reporter cell lines were established in a similar way than HELN-hERα and -hERβ cell lines (Pinto et al., 2014). Briefly, HELN-ERs cell lines cells were obtained by transfection of HELN cells (HeLa cells stably transfected with the ERE-β Globin-Luc-SVNeo plasmid) (Balaguer et al., 1999) by the corresponding pSG5-puro plasmids (pSG5-hERα-puro, -hERβ-puro -zfERα-puro, -zfERβ1-puro, and -zfERβ2-puro, respectively).

## Selectivity of chemicals for human and zebrafish estrogen receptors

Screening of endogenous, environmental and synthetic ligands in the HELN-zfER cell lines showed that known mammalian ER ligands are also able to induce transcriptional activity of zebrafish ER subtypes (Pinto et al., 2014). This screening allowed us to assess differences in the potency of the estrogenic compounds among the three zfER subtypes, and compare their selectivity toward hERs using a similar human cellular context. The HELN-zfERs cells were incubated at 28°C after addition of chemicals to the cells because it is a more physiologically relevant temperature for zebrafish, which increased the potency of estradiol approximately 10-fold compared to incubation at 37°C. Temperature sensitivity of fish ERs has already been reported using reporter gene assays (Matthews et al., 2002; Cosnefroy et al., 2009) and the reason seems to be thermo-dependence of estrogen binding (Tan et al., 1999; Matthews et al., 2002; Sumida et al., 2003).

We have shown that there are clear differences between the selectivity of various (anti)estrogens for zebrafish and human ER isoforms, establishing the fact that a direct translation of (anti)estrogenic effects (activities or potencies) from mammals to zebrafish is not possible. Although none of the tested compounds specifically activated either zebrafish or human ERs, transcriptional activities toward human and zebrafish ERs need to be studied.

**Figure 1:**
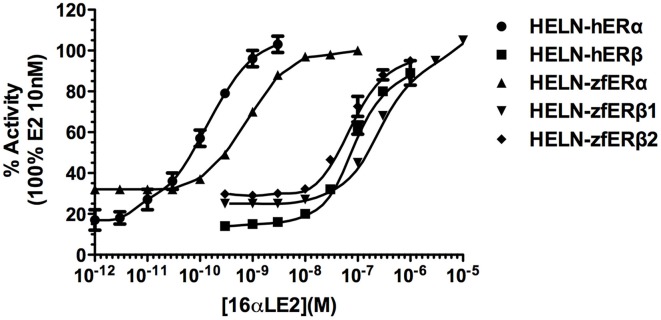
**Transcriptional activity of hERα, hERβ, zfERα, zfERβ1, and zfERβ2 in response to the synthetic pharmaceutical compound 16α-LE2.** HELN-hERα (●), -hERβ1 (■), -zfERα (○), HELN-zfERβ1 (□), HELN-zfERβ2 (◊) cells were exposed to different concentrations of 16α-LE2. Results are expressed as % of 10 nM E2 treatment and are derived from Escande et al. (2006) and Pinto et al. (2014).

Natural (E2) and pharmaceutical (EE2) estrogens display similar affinities for hERs and zfERs. Some environmental estrogens (α-zearalanol, bisphenol-A) with similar affinity for hERs preferentially activated zfERα rather than zfERβ s. Other environmental estrogens (nonylphenol mixture, 4-tert-octylphenol) with similar affinity for hERs displayed slightly higher affinity for zfERα and zfERβ2 than for zfERβ1. Benzophenone 2 and phytoestrogens (genistein, liquiritigenin) which have higher affinity for hERβ than for hERα also displayed slightly higher affinity for zfERα and zfERβ2 than for zfERβ1. Finally, hERβ selective synthetic compounds (8β-vE2, DPN) preferentially activated zfERα compared to zfERβs. On the contrary and similar to hERs, the synthetic compound 16α-E2, which has 1000-fold more selectivity for hERα (Escande et al., 2006), also exhibited higher affinity for zfERα compared to the zfERβ subtypes and is the most selective compound for zfERα nowadays (Figure 1; Table 1).

**Table 1:** **16αLE2 ERs EC_50_ and maximal activities**.

**NR**	**EC_50_ (nM)**	**Maximal activity (%)**
hERα	0.093 ± 0.025	100
hERβ	92.9 ± 1.4	100
zfERα	0.79 ± 0.43	100
zfERβ1	240 ± 30.2	100
zfERβ2	74.6 ± 7.08	100

**Figure 2:**
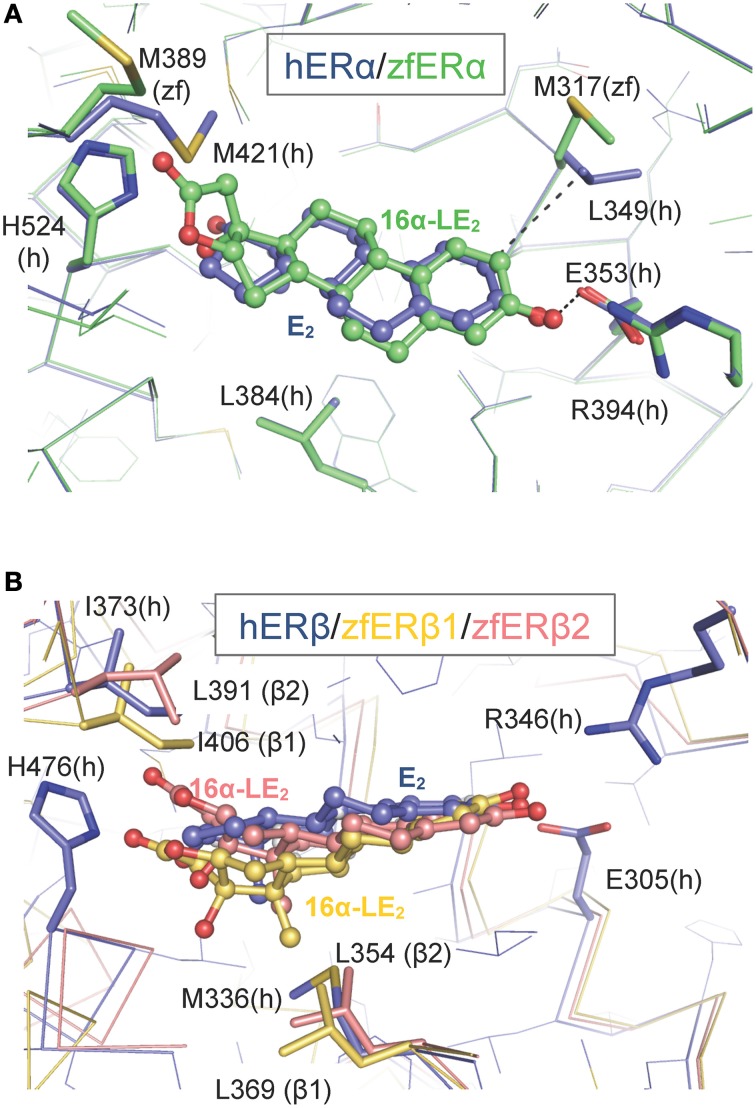
**Modeling of the interaction between 16α-LE2 and the human (h) and zebrafish (zf) estrogen receptors.** Superposition of the structures of hERα **(A)** and hERβ **(B)** LBDs bound to E2 (blue) on to the molecular models of the 16α-LE2-bound zfERα **(A)** (green) and 16α-LE2-bound hERβ1 (red) and 16α-LE2-bound hERβ2 (yellow) **(B)** LBDs. In zfERα, the lactone ring of 16α-LE2 points toward M421 (H7) which undergoes a large conformational change (black arrow) to accommodate this additional group. In hERβ, the linear M421 present in ERα (M389 in zfERα) is replaced by branched residues (I373 in hERβ, I406 in zfERβ1 and L391 in zfERβ2), which are characterized by a much smaller intrinsic flexibility that maintains the synthetic ligand in a position where it interacts unfavorably with M336 in hERβ, L369 in zfERβ1 and L354 in zfERβ2. This figure is derived from Pinto et al. (2014).

To gain structural insights into the zfERα selectivity of 16α-LE2 in human and zebrafish ERs, we used the web-based server EDMon (Endocrine Disruptor Monitoring; http://atome2.cbs.cnrs.fr/AT2B/SERVER/EDMon.html) (Delfosse et al., 2012) to model zfERs in complex with this ligand. The structural basis of the hERα and hERβ selectivity toward certain ligands has been associated with two amino acid differences in their ligand-binding pockets. Indeed, L384 and M421 of hERα are replaced by M336 and I373 in hERβ, respectively (Figure 2 and Manas et al., 2004). Superimposition of the 16α-LE2-bound zfERα model on the crystal structure of hERα in complex with E2 (PDB code 3UUD) showed that the phenol ring of 16α-LE2 occupies the same position as that of E2 and is engaged in a network of hydrogen bonds with E353 from helix 3 (H3) and R394 from H5 (Figure 2A). On the other side of the ligand-binding pocket (LBP), it appears that the hydrogen bond observed between the 17-hydroxyl group of E2 and H524 (H11) is conserved in 16α-LE2. The difference between the two complexes resides in the lactone ring of 16α-LE2 which points toward M421 (H7) that must undergo a large conformational change to accommodate this additional group. In hERβ, the linear M421 is replaced by the branched residue Ileu 373 characterized by a much smaller intrinsic flexibility (Figure 2B). As a consequence, I373 maintains the synthetic ligand in a position where it interacts unfavorably with M336 (H3). Therefore, 16α-LE2 adopts different positions in hERα and hERβ, the more constrained environment provided by the latter accounting for the weaker affinity of the ligand for this receptor subtype. The affinity values measured with the zebrafish receptors reflect the variations in the space constraints provided by the different combinations of residues in the three receptor subtypes. With H3 and H7 residues identical to those of the human receptor, zfERα interacts with 16α-LE2 with the highest affinity. The slight difference in the binding affinity of 16α-LE2 for hERα and zfERα relies most likely on the replacement of L349 (H3) by a methionine residue (M317) (Figure 2A) and a possible loss of a favorable interaction provided by the branched but not by the linear residue (Figure 2A). With a conserved isoleucine in H7 (I406) and a leucine residue in H3 (L369) (Figure 2B), zfERβ1 displays the most constrained LBP reflecting the weakest binding affinity for 16α-LE2. This receptor combines two large residues with low (isoleucine) and medium (leucine) flexibilities. The replacement of I406 in H7 of zfERβ1 by a leucine residue (L391) (Figure 2B) in zfERβ2 provides a slight gain in LBP plasticity, in agreement with the slightly better affinity of 16α-LE2 for the latter.

The inability of hERβ-selective phytoestrogens (genistein and liquiritigenin) and pharmaceuticals (8bv-E2, DPN) to activate preferentially the zfERβ isoforms is explained by the mutation of a critical amino acid involved in genistein binding in hERβ. In all zfERs, the position homologous to hERβ M336 is occupied, as in hERα, by a leucine residue (Figure 2) (Sassi-Messai et al., 2009). This amino acid change most likely accounts for the lack of obvious selectivity of the phytoestrogens toward the zfERβ subtypes.

## Peroxysome proliferator γ

PPARs are involved in the regulation of glucose, lipid, and cholesterol metabolism in response to fatty acids and their derivatives, eicosanoids, and drugs used in the treatment of hyperlipidemia and diabetes. The human PPAR subfamily contains three members known as hPPARα, hPPARβ, and hPPARγ. Each hPPAR subtype shows a distinct tissue distribution and ligand preference. hPPARγ is highly expressed in adipose tissue and is a central regulator of lipid storage and adipocyte gene expression and differentiation (Tontonoz et al., 1995) and is involved in various pathophysiological disorders, including metabolic disease, insulin resistance, and diabetes (Rosen and Spiegelman, 2001). hPPARγ is the target for antidiabetic agents of the thiazolidinedione class, which includes troglitazone, pioglitazone, and rosiglitazone. The LBD of hPPARγ is rather large and the diversity of ligands that can be accommodated within its pocket, mainly represented by lipid derivatives, may contribute to the large array of roles that have been assigned to hPPARγ. Given the physiological role of hPPARγ in adipose tissue development and maintenance, it has been proposed that disruption of regulation pathways under the control of hPPARγ may be involved in the onset of diabetes and obesity (Swedenborg et al., 2009). Indeed, activation of this receptor by certain xenobiotic compounds has been shown to stimulate adipogenesis *in vitro* and *in vivo* through induction of the differentiation of preadipocytes of the fibroblastic lineage into mature adipocytes (Grun and Blumberg, 2009; le Maire et al., 2009; Janesick and Blumberg, 2011; Riu et al., 2011a). This has led to the “obesogen hypothesis,” according to which, in addition to disruption of the balance between caloric intake and expenditure characterizing modern life-style, the rapidly growing obesity epidemic could also implicate environmental risk factors including an increased exposure to chemicals that interfere with any aspects of metabolism (Grun and Blumberg, 2009; Janesick and Blumberg, 2011, 2012). Accordingly, compounds that have the potential to disrupt any metabolic signaling pathways and lead to increased fat accumulation and obesity are referred to as “obesogens” (Grun and Blumberg, 2006).

Like for ERs, zebrafish begin to be used as model species to study *in vivo* effects of EDCs on PPARγ (Lyche et al., 2011; Riu et al., 2014). Similar to mammals, zebrafish store neutral lipid triglycerides in the visceral, intramuscular, and subcutaneous adipocyte depots. The first adipocytes, which can be observed from day 8 to 12, or at a minimal size of about 5 mm (Imrie and Sadler, 2010), appear in the pancreatic region, then in the viscera, and later on, in the subcutaneous and cranial regions (Flynn et al., 2009; Imrie and Sadler, 2010). Lipid staining can be detected before this stage; however, at this time point, the lipids are not stored in adipocytes, but rather in the yolk, hepatocytes, blood vessels, skeletal myocytes, jaw chondrocytes, and neuronal tissue in the brain (Imrie and Sadler, 2010).

## Pparγ reporter cell lines

HG5LN-hPPARγ and -zfPPARγ reporter cell lines were established in a similar way (Seimandi et al., 2005; Riu et al., 2014). Briefly, HG5LN-PPARγ cell line was obtained by transfection of HG5LN cells (HeLa cells stably transfected with the GALRE5-β Globin-Luc-SVNeo plasmid) (Seimandi et al., 2005) by the corresponding pSG5-puro plasmids [pSG5-GAL4(DBD)-hPPARγ(LBD)-puro and -zfPPARγ(LBD)-puro, respectively]. Interestingly, the thermodependence observed for zfERs is not shared by zfPPARγ (Riu et al., 2014).

## Selectivity of chemicals for human and zebrafish PPARγ

**Figure 3:**
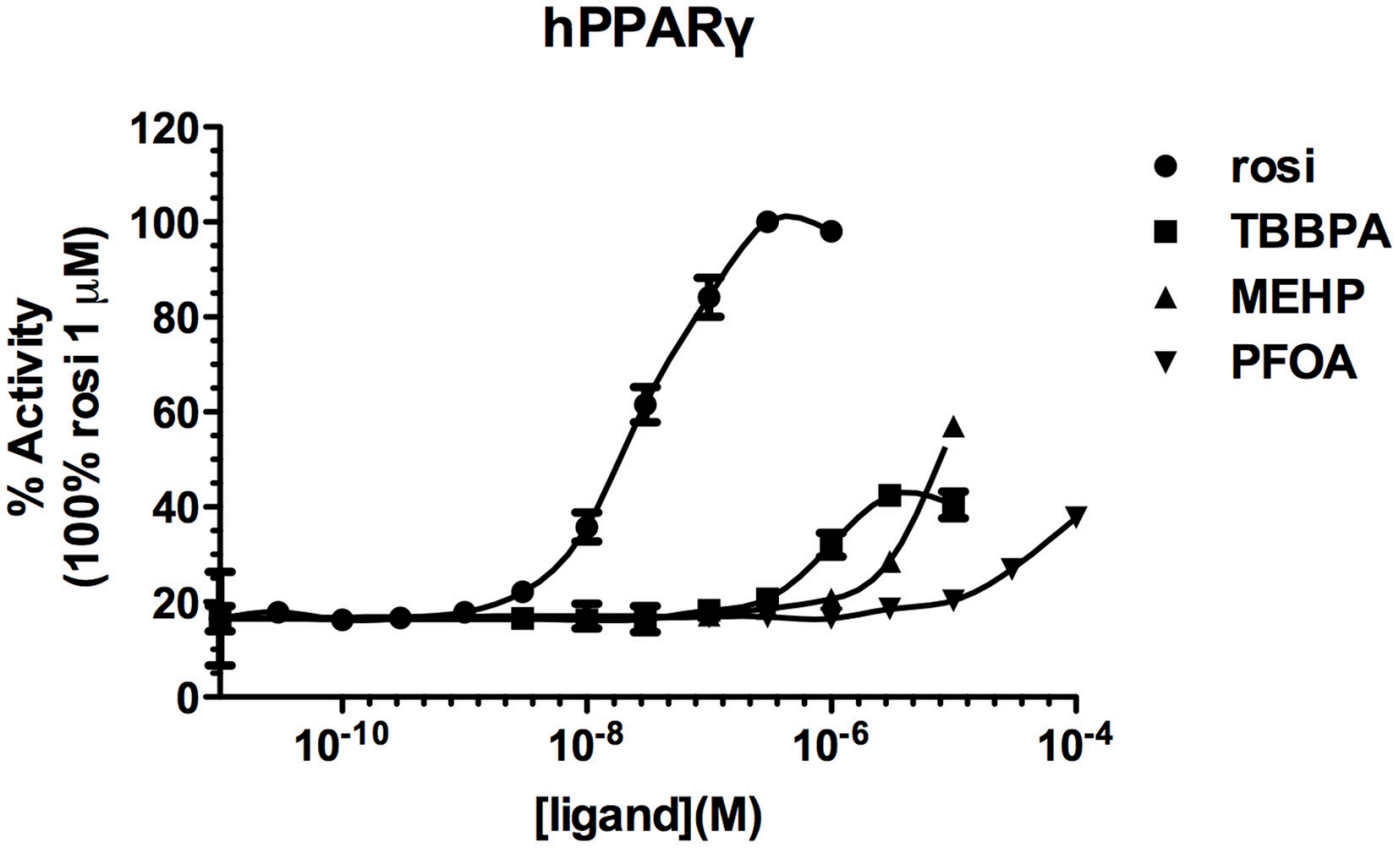
**Transcriptional activity of hPPARγ in response to the synthetic pharmaceutical compound rosiglitazone and the environmental compounds TBBPA, MEHP, and PFOA.** HG5LN hPPARγ and zfPPARγ cells were exposed to different concentrations of rosiglitazone (●), TBBPA (□), MEHP (○), and PFOA (◊). Results are expressed as % of basal activity and are derived from Riu et al. (2011a).

**Figure 4:**
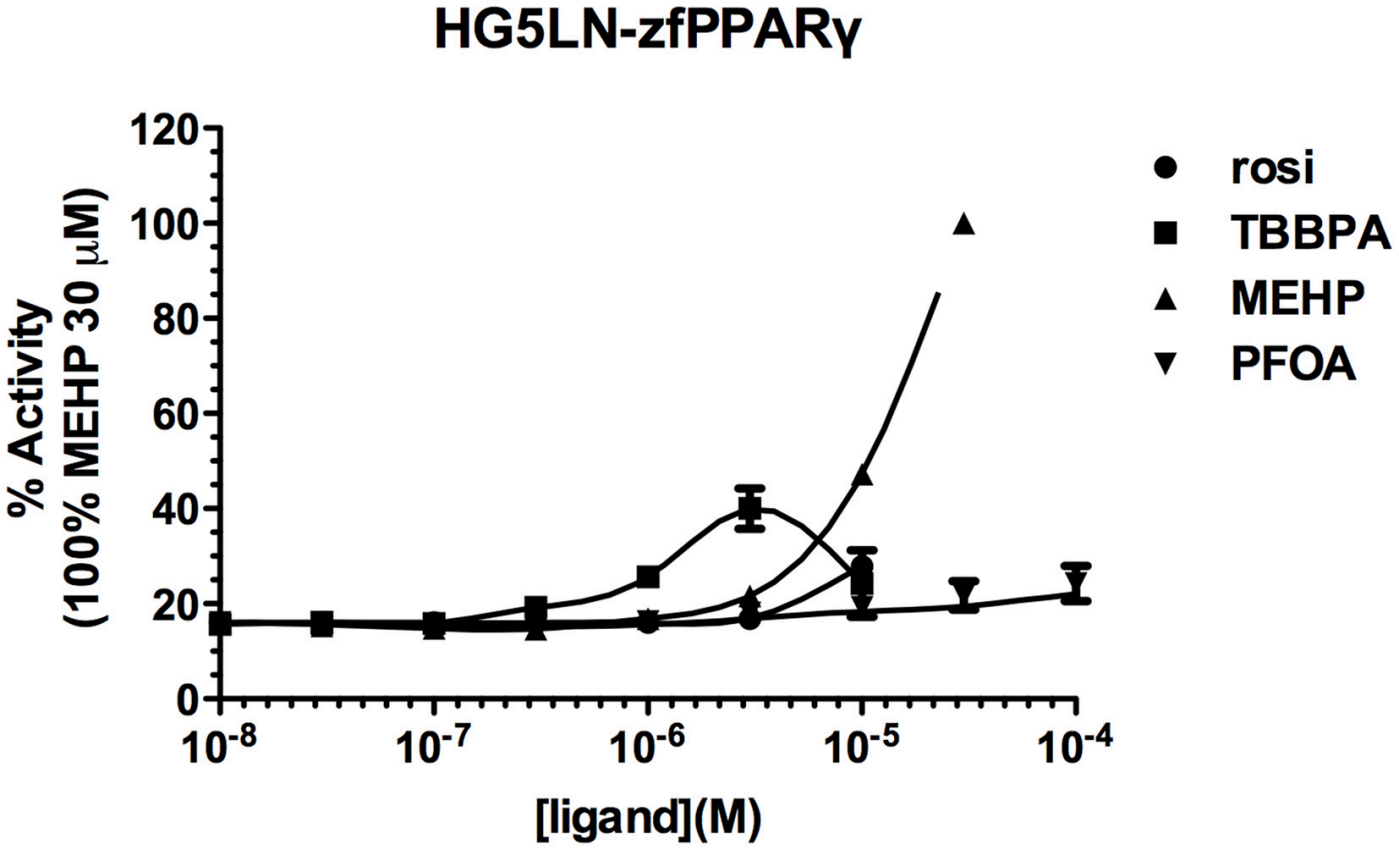
**Transcriptional activity of zfPPARγ in response to the synthetic pharmaceutical compound rosiglitazone and the environmental compounds TBBPA, MEHP, and PFOA.** HG5LN hPPARγ and zfPPARγ cells were exposed to different concentrations of rosiglitazone (●), TBBPA (□), MEHP (○), and PFOA (◊). Results are expressed as % of basal activity and are derived from Riu et al. (2011a) and Riu et al. (2014).

Screening of environmental and pharmaceutical ligands in the HG5LN-zfPPARγ cell lines showed that known hPPARγ ligands are not always able to induce transcriptional activity of zebrafish PPARγ (Riu et al., 2011a). Pharmaceutical hPPARγ ligands like thiazolidones (rosiglitazone, troglitazone) do not or very weakly bind to zfPPARγ. On the contrary, environmental PPARγ compounds including phthalates (MEHP), perfluorinated compounds (PFOA, PFOS) and halogenated derivatives of BPA (TBBPA, TCBPA) are common activators of hPPARγ and zfPPARγ (Figures 3, 4; Table 2). We also provide evidence that activation of ER**s** and PPAR**γ** depends on the halogenation degree of BPA analogs. The bulkier are brominated BPA analogs, the greater is their capability to activate PPAR**γ** and the weaker is their estrogenic potential (Riu et al., 2011b).

**Table 2:** **EC_50_ and maximal activities of PPARγ ligands**.

	**hPPARγ**	**zfPPARγ**
**Ligand**	**EC_50_ (μM) (maximal activity %)**	**E_50_ (μM) (maximal activity %)**
Rosiglitazone	0.027 ± 0.003 (100)	ND (27.9)
TBBPA	0.762 ± 0.136 (42,4)	1.45 ± 0.33 (40)
MEHP	1050 ± 73 (57)	11.3 ± 1.29 (100)
PFOA	380 ± 71 (37.8)	ND (24.2)

ND, Not determined.

**Figure 5:**
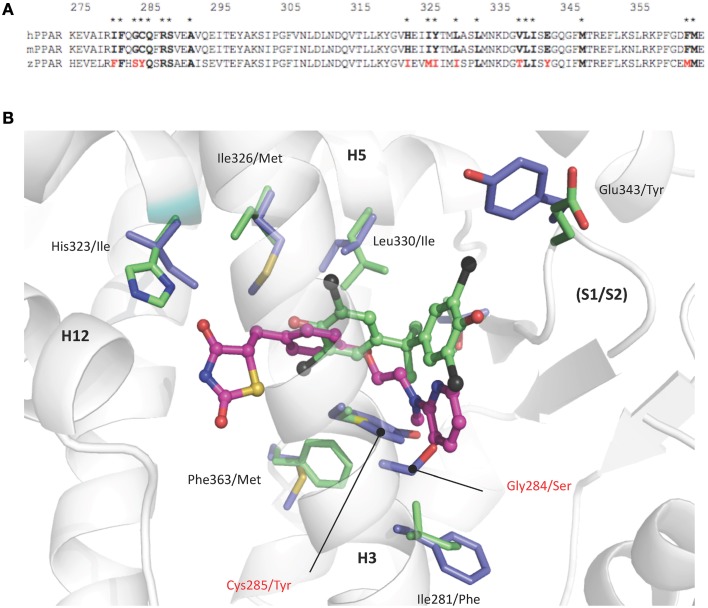
**(A) Sequence alignment of human, mouse, and zebrafish PPARγ ligand binding pocket residues.** Asterisks denote residues in contact with TBBPA and/or rosiglitazone (PDB code 2PRG). Interacting residues that differ between sequences are highlighted in red. **(B)** TBBPA (carbon atoms colored in green) and rosiglitazone (magenta, PDB code 2PRG) as they are positioned in the human PPARγ. Residues that differ in the ligand binding pocket of human and zebrafish PPARγ are displayed as green and blue sticks, respectively. The human PPARγ Gly284 and Cys285 which are replaced by serine and tyrosine residues in zebrafish PPARγ are indicated in red. This figure is derived from Riu et al. (2011a).

Comparison of human and zebrafish PPARγ sequences reveals several residue differences which could explain the differential ligand specificity of the various species (Figure 5A). In particular, the replacement of human PPARγ Gly284 and Cys285 by serine and tyrosine residues in zebrafish PPARγ provides a rationale for the weak binding affinity of rosiglitazone for this receptor as compared to that observed for the human homolog (Figure 4B). In contrast, the different binding mode of halogenated compounds allows both hPPARγ and zfPPARγ to accommodate TBBPA and TCBPA (Figure 5B).

Structural and biophysical studies revealed that TBT binds to both hRXR and hPPARγ through formation of a covalent bond between the tin atom and the sulfur atom of cysteine residues located in the LBP of both receptors (le Maire et al., 2009; Delfosse et al., 2014). In RXR, this cysteine (Cys432) is located in helix H11 and is conserved in several species. In contrast, the cysteine residue of PPARγ (Cys285) resides in H3 and is not conserved in several species including zebrafish.

## Conclusion

We have shown above that there are clear differences between the activity of various EDCs for zebrafish and human ERs and PPARs, demonstrating that a direct translation of effects from mammals to zebrafish is not possible. The differences revealed in this study, in terms of transcriptional activities toward human and zebrafish ERs and PPARs, highlight the need to take into account the species of origin when assessing the potency of chemicals. This is particularly important with regard to EDCs screening for hazard assessment since at the present time established test guidelines are only based on human cell lines expressing human nuclear receptors.

To this end, such *in vitro* cell lines expressing zebrafish nuclear receptors can serve as useful screening tools to address nuclear receptor potency of chemicals for fish models. Hence, an initial screening should be followed up with an NR-subtype specific analysis using both human and zebrafish NRs to elucidate the full spectrum of NR-mediated EDCs effects.

### Conflict of interest statement

The authors declare that the research was conducted in the absence of any commercial or financial relationships that could be construed as a potential conflict of interest.
